# Connectivity-based segmentation of the thalamic motor region for deep brain stimulation in essential tremor: A comparison of deterministic and probabilistic tractography

**DOI:** 10.1016/j.nicl.2024.103587

**Published:** 2024-02-27

**Authors:** Evangelia Tsolaki, Alon Kashanian, Kevin Chiu, Ausaf Bari, Nader Pouratian

**Affiliations:** a Department of Neurosurgery, David Geffen School of Medicine at UCLA, Los Angeles, CA, USA; b Department of Neurosurgery, Donald and Barbara Zucker School of Medicine at Hofstra/Northwell, Hempstead, NY, USA; c Brainlab, Inc., 5 Westbrook Corporate Center, Suite 1000, Westchester, IL 60154, USA; d Department of Neurological Surgery, UT Southwestern Medical Center, Dallas, TX, USA

**Keywords:** Deep brain stimulation, Deterministic, Diffusion tractography, Essential tremor, Probabilistic, Thalamus

## Abstract

**Objective:**

Deep brain stimulation (DBS) studies have shown that stimulation of the motor segment of the thalamus based on probabilistic tractography is predictive of improvement in essential tremor (ET). However, probabilistic methods are computationally demanding, requiring the need for alternative tractography methods for use in the clinical setting. The purpose of this study was to compare probabilistic vs deterministic tractography methods for connectivity-based targeting in patients with ET.

**Methods:**

Probabilistic and deterministic tractography methods were retrospectively applied to diffusion-weighted data sets in 36 patients with refractory ET. The thalamus and precentral gyrus were selected as regions of interest and fiber tracking was performed between these regions to produce connectivity-based thalamic segmentations, per prior methods. The resultant deterministic target maps were compared with those of thresholded probabilistic maps. The center of gravity (CG) of each connectivity map was determined and the differences in spatial distribution between the tractography methods were characterized. Furthermore, the intersection between the connectivity maps and CGs with the therapeutic volume of tissue activated (VTA) was calculated. A mixed linear model was then used to assess clinical improvement in tremor with volume of overlap.

**Results:**

Both tractography methods delineated the region of the thalamus with connectivity to the precentral gyrus to be within the posterolateral aspect of the thalamus. The average CG of deterministic maps was more medial-posterior in both the left (3.7 ± 1.3 mm^3^) and the right (3.5 ± 2.2 mm^3^) hemispheres when compared to 30 %-thresholded probabilistic maps. Mixed linear model showed that the volume of overlap between CGs of deterministic and probabilistic targeting maps and therapeutic VTAs were significant predictors of clinical improvement.

**Conclusions:**

Deterministic tractography can reconstruct DBS thalamic target maps in approximately 5 min comparable to those produced by probabilistic methods that require > 12 h to generate. Despite differences in CG between the methods, both deterministic-based and probabilistic targeting were predictive of clinical improvement in ET.

## Highlights

• Comparison of tractography methods for connectivity-based DBS targeting in ET patients.
• Both methods defined the thalamic posterolateral area to be connected to precentral gyrus.
• Deterministic maps were found more medial posterior compared to probabilistic maps.
• Both methods can reconstruct comparable thalamic target maps for DBS.

## 1 Introduction

Deep brain stimulation (DBS) is an established therapy for medically refractory movement disorders including Parkinson disease (PD), essential tremor (ET), and dystonia, and is currently under investigation for several other neurologic and psychiatric conditions (Lozano et al., 2019). White matter connections may play an important role in mediating the therapeutic effect of DBS, as has been implicated by a number of studies comparing diffusion tractography-derived white matter tracts and DBS outcomes as well as animal studies pointing towards modulation of white matter projections (Calabrese, 2016, Gradinaru et al., 2009). Diffusion tractography (DT) provides a non-invasive method to indirectly visualize white matter pathways in-vivo based on patterns of water diffusivity, leading many to advocate its use to guide DBS targeting. By delineating underlying white matter tracts, DT can help inform DBS target selection in a personalized manner. There have been numerous retrospective neuroimaging studies over the past decade which have demonstrated the potential of DT to optimize clinical outcomes through connectivity-based targeting (Calabrese, 2016, Middlebrooks et al., 2020, Gravbrot et al., 2020). This has been particularly useful for DBS and high-intensity focused ultrasound for ET, where the most common brain target, the ventral intermediate nucleus (Vim), is difficult to visualize on MRI and is traditionally targeted indirectly (Gravbrot et al., 2020, Tsolaki et al., 2018). Rather than relying on atlas-defined coordinates, our group and others have demonstrated that DT can be used to identify patient-specific therapeutic thalamic targets based on individualized thalamic connectivity patterns (Tsolaki et al., 2018, Pouratian et al., 2011, Middlebrooks et al., 2018, Behrens et al., 2003, Riskin-Jones et al., 2021). Personalized DBS targeting with DT may offer potential benefits including improved targeting with better outcomes and less adverse events. See (Table 1).

**Table 1:** Demographic data.

**Subject**	**Age**	**Sex**	**Site**	**CRTS** **Pre-L**	**CRTS** **Op L**	**CRTS % L**	**CRTS** **Pre-R**	**CRTS** **Op R**	**CRTS % R**
S_01	46	F	Unilateral						
S_04	67	F	Unilateral	17	1	94			
S_05	77	M	Unilateral						
S_06	45	F	Unilateral	12	4	67			
S_07	72	F	Unilateral	17	5	71			
S_08	78	M	Unilateral						
S_11	77	M	Bilateral	16	6	63	20	19	5
S_13	63	F	Bilateral						
S_14	60	F	Bilateral	28	9	68	19	0	100
S_15	61	F	Bilateral	23	2	91	21	17	19
S_16	69	F	Bilateral	25	6	76			
S_17	68	M	Bilateral	12	7	42	13	6	54
S_18	46	F	Bilateral	14	7	50	24	11	54
S_19	58	M	Bilateral						
S_20	64	M	Unilateral						
S_21	61	F	Bilateral	28	6	79	10	6	40
S_22	83	M	Unilateral						
S_24	61	M	Bilateral	14	2	86	23	5	78
S_25	72	F	Bilateral						
S_26	82	F	Unilateral				22	7	68
S_29	75	F	Unilateral						
S_30	72	F	Unilateral	14	2	86			
S_31	65	M	Bilateral	19	7	63	16	6	63
S_35	68	F	Unilateral						
S_36	71	F	Bilateral	18	8	56	13	8	38
S_37	80	F	Bilateral	13	6	54	12	10	17
S_39	75	F	Unilateral						
S_40	68	M	Bilateral						
S_41	78	F	Unilateral						
S_42	66	F	Bilateral						
S_43	67	F	Bilateral						
S_44	75	M	Unilateral	28	4	86			
S_45	75	F	Bilateral						
S_46	71	M	Bilateral	24	0	100	15	4	73
S_47	73	M	Bilateral	11	5	55	13	6	54
S_48	72	M	Unilateral						

Ideally, the incorporation of DT-based targeting into DBS surgical planning should involve methods that are both neuroanatomically accurate and practical to implement. Tractography methods can be broadly classified into two algorithms: deterministic and probabilistic. Deterministic tractography models a single principal fiber orientation at each voxel, and thereby produces only one streamline per seed voxel. A major criticism of this method is that it is unable to accurately represent fiber configurations in voxels containing multiple crossing-fiber populations. In contrast, probabilistic tractography estimates a distribution of fiber orientations for each voxel and randomly draws from this distribution to produce multiple streamline samples per seed voxel (Gravbrot et al., 2020). For this reason, probabilistic methods can better account for uncertainty in data and are considered more sensitive than deterministic methods for modeling non-dominant fiber pathways (Behrens et al., 2007). However, this random iterative process is computationally demanding, time consuming, and therefore, largely remains a research tool. It may also be unnecessary for modeling certain pathways, such as the thalamocortical system in which crossing fibers may not be as significant a concern. Conversely, deterministic tractography demands little to no training, requires considerably less processing time, and is already integrated into several commercially available neuronavigation systems. Despite being considered generally less suitable to model complex fiber pathways, deterministic algorithms can outperform their probabilistic counterparts in some cases (Maier-Hein et al., 2017). Depending on the pathway of interest, deterministic methods may be sufficient for DBS application.

The tractography methods used to target the thalamus for tremor have been discrepant across studies with similar yet distinct reports of clinically effective connectivity patterns (Riskin-Jones et al., 2021, Gravbrot et al., 2020, Tsolaki et al., 2018, Pouratian et al., 2011, Middlebrooks et al., 2018, Coenen et al., 2014, JM, 2015, Schlaier et al., 2015, Fenoy, 2017, Fenoy and Schiess, 2018, King et al., 2017, Sasada et al., 2017, Chazen et al., 2017, Nowacki et al., 2018, Elias et al., 2012, Sudhyadhom et al., 2013, Klein et al., 2012, Akram et al., 2018, Anthofer et al., 2014). While differences in targeting based on tractography methodology have been evaluated for the STN, such a comparison for thalamic targeting has been limited (Schlaier et al., 2017, Yang et al., 2022). Addressing this critical gap in knowledge may help explain the relative similarity in results across methodologies, with our underlying hypothesis being that the two methods will generally produce similar targeting results. On the other hand, differences that may be uncovered by direct comparisons may also shed light on reported discrepancies across studies. Amongst the approaches that have been described for using tractography to target the optimal region of the thalamus for tremor control, one of those methods involves using probabilistic tractography to target the region of the thalamus with highest connectivity to the precentral gyrus (Pouratian et al., 2011, Middlebrooks et al., 2018). However, it remains unclear whether deterministic methods that are used in clinical practice, such as BrainLab software, mirror probabilistic approaches that have been used and published to assess thalamic targeting (i.e., FSL) so as to interchange techniques and extrapolate results from one method to the other. The objective of this study was to compare deterministic and probabilistic tractography methods for connectivity-based targeting in ET patients by retrospectively comparing thalamic segmentations produced by each method in individuals with ET.

## 2 Methods

### 2.1 Participants

This is a retrospective single-center study of 36 patients (14 males and 22 females, Age: 68.3 ± 9.4 (standard deviation) years) with ET who underwent stereotactic implantation of DBS electrodes and had diffusion weighted imaging (DWI) sequences included in their preoperative MRI scan for DBS planning. Patient age ranged from 42 to 82 years. Only patients with ET were included in the current analysis to avoid potential disease-specific differences in white matter integrity and imaging characteristics that could complicate interpretation. All patients had a diagnosis of ET according to standard clinical criteria. None of the patients had other neurologic comorbidities. The study was approved by our local Institutional Review Board. Tremor measurements using the Clinical Rating Scale for Tremor (CRST) (Jankovic and Tolosa, 2007) questionnaire before (within 3 months prior to surgery) and after DBS (approximately 6 months after initial programming, per clinical routine) were available in a subset of patients (N = 18, 6: unilateral DBS, 12: bilateral DBS). Tremor scores ranged from 0 to 32 and were derived from Parts A and B of the CRST, in the hand contralateral to the site of brain stimulation.

### 2.2 Image acquisition

All patients underwent preoperative 3 T MRI imaging on a Siemens Skyra (n = 20), Prisma (n = 9), Sensation (n = 5) or Trio (n = 2) scanner prior to DBS surgery. High resolution T1-weighted anatomical images based on magnetization prepared rapid acquisition gradient echo (MPRAGE) sequences were acquired using the following parameters: TE = 2.44 or 2.98 ms, TR = 2.1 or 2.5 s, matrix = 256 x 256, isotropic 1 mm voxels, and flip angle = 9 or 15°. Single shot spin echo planar imaging for DWI were acquired with the following parameters: TE = 66, 72, 74, 75, or 91 ms, TR = 7, 7.1, 7.2, or 7.6 s, matrix = 128 x 128, voxel size = 1.7 x 1.7 mm or 2 x 2 mm, slice thickness = 2 or 3 mm, b-value = 1000, and 64 directions.

### 2.3 Calculation of volume of tissue activation

The SimBio/Fieldtrip model (Horn et al., 2017) in Lead DBS v2.1 (Horn and Kühn, 2015) (https://www.lead-dbs.org) was used to calculate the volume of tissue activated (VTA) for each patient’s final therapeutic programming configuration. The VTAs were calculated using post-operative CT scans in native patient space as described in our previous study (Riskin-Jones et al., 2021). As lead-DBS resamples the images and VTAs to a higher resolution for modeling, the post-op CT scans and VTAs were resampled and co-registered back to raw T1 space using SPM version 12 (nearest neighbor interpolation, no wrap) (Friston et al., 1995), then registered to diffusion tensor imaging (DTI) space using the linear registration FLIRT (6 degrees of freedom) tool in FMRIB Software Library (FSL) (Jenkinson et al., 2002, Jenkinson and Smith, 2001).

### 2.4 Probabilistic tractography - Preprocessing

A similar version of the tractography preprocessing pipeline was previously described by Tsolaki et al., (Tsolaki et al., 2018). Eddy current correction was performed by registering each volume in the diffusion data set to the initial B0 volume using an affine transformation. Skull stripping was performed using the brain extraction tool (BET) (Smith, 2002). A multi-fiber diffusion model in FDT was fitted on the data (Behrens et al., 2003). This model uses Bayesian techniques to estimate a probability distribution function (PDF) on the principal fiber direction at each voxel, accounting for the possibility of crossing fibers within each voxel. Three fibers were modeled per voxel, a multiplicative factor (i.e., weight) of 1 for the prior on the additional modeled fibers, and 1000 iterations before sampling (Behrens et al., 2007). FreeSurfer (version 5.3.0, https://surfer.nmr.mgh.harvard.edu/) (Fischl et al., 1999, Dale et al., 1999) was used to automatically delineate the primary motor cortex and as well as thalamic regions of interest (ROIs) on pre-treatment T1-weighted images for each individual. The T1-weighted structural images were registered to DTI space using linear registration in FLIRT (6 degrees of freedom) (Jenkinson and Smith, 2001, Jenkinson et al., 2002). ROIs were then registered to DTI space according to the same transformation matrix.

### 2.5 Probabilistic tractography analysis

**Fig. 1:**
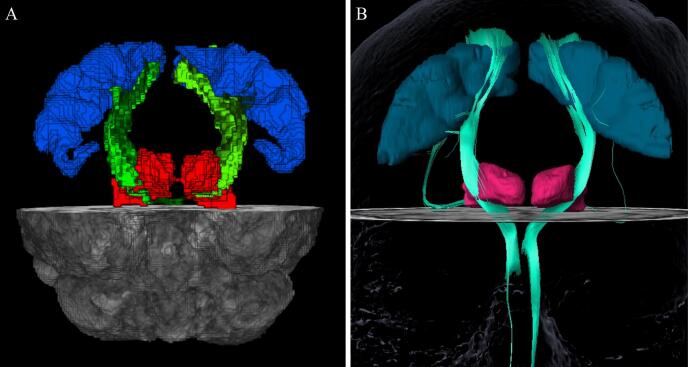
Tractography Methods. Three-Dimensional tractography results from a single patient in T1-weighted space demonstrating streamlines (green) between the thalamus (red) and precentral gyrus (blue) using the probabilistic (A) and deterministic methods (B). Three-dimensional reconstructions of probabilistic and deterministic fiber tracts were created using MRtrix (https://www.mrtrix.org) and Brainlab, respectively. (For interpretation of the references to color in this figure legend, the reader is referred to the web version of this article.)

Probabilistic tractography was performed to define the structural connectivity between the thalamus and the primary motor cortex using FMRIB's Software Library (FSL) on a system with GPU computing (Fig. 1**A**). Using the PDFs and PROBTRACKX, we determined the probability of connectivity between the thalamus and primary motor cortex. From each voxel in the thalamic seed, 5000 streamlines were generated; a 0.2 curvature threshold was chosen, a loop check termination was used, and the target masks were used as waypoint, termination, and classification masks. The resulting tomographic probabilistic maps identified the regions within thalamus with the highest probability of connectivity with the primary motor cortex. To define the area within the thalamus with high probabilistic of connectivity to precentral gyrus, different thresholds were applied to the resulting thalamic parcellation maps. To ensure robustness of results, multiple thresholds were evaluated. Thresholds were based on the maximum intensity within each segmented thalamic map acting as “high pass” filters allowing only the voxels with higher connectivity to the target to “pass”. The ideal threshold is unknown, so varying thresholds of 30–50 % of the maximum intensity value were applied as has been described previously to identify the areas within the thalamic maps with higher probability of connectivity to the precentral gyrus (Tsolaki et al., 2018).

### 2.6 Deterministic tractography - Preprocessing

The deterministic tractography processing pipeline was fully performed with the Brainlab Elements Fibertracking software suite (Brainlab, Munich, Germany, version 1.0.0.113) (Fig. 1**B**). A “Cranial Planning” workflow was selected, and individual patient DICOM imaging data were imported, including high resolution T1-weighted structural and diffusion sequences. Eddy current distortions were corrected, and DTI sequences were fused with the T1 sequences by an automatic rigid registration. Thalamus and precentral gyrus ROIs were segmented automatically using Brainlab Elements Segmentation Cranial (version 5.0.0.72) and Brainlab Elements Object Manipulation (version 1.1.1.8).

### 2.7 Deterministic tractography analysis

The thalamus and precentral gyrus ROIs were selected as seed regions and fiber tracking was performed using Brainlab’s default fiber tracking parameters: minimum fractional anisotropy (FA) = 0.20, minimum fiber length = 80 mm, and maximum fiber bundle angulation = 20°. Brainlab’s fiber tracking algorithm is based on a local diffusion approach called FACT (fiber assignment by continuous tracking) (Mori and Van Zijl, 2002). The effect of the tracking is a three-dimensional parametric display of fibers passing through the defined ROIs. The resultant thalamus-to-precentral gyrus tract was then converted into an object, and the intersecting volume between this tract and the thalamus was created using Brainlab’s “Intersection” function. Hence, this new intersecting volume represented the region of the thalamus with connectivity to the precentral gyrus, as determined by the deterministic tracking algorithm.

### 2.8 Comparing deterministic and probabilistic segmentation maps

For our qualitative comparison between deterministic and probabilistic thalamic segmentation maps, first deterministic thalamic segmentation maps were exported from the Brainlab software and converted from DICOM to NIFTI format using 3D Slicer (version 4.10.2, https://www.slicer.org) in order to import them into the FSL environment for comparison with probabilistic tractography. Then, each individual’s deterministic map was co-registered to their respective T1 native space using SPM version 12 (nearest neighbor interpolation, no wrap) (Friston et al., 1995). After observing general agreement between the deterministic and probabilistic maps, a quantitative comparison of volume of overlap was conducted by finding the intersection between deterministic and probabilistic maps with 30, 40, and 50 % thresholds (DP30, DP40, DP50) to ensure robustness of results and that results were not a result of selection of an arbitrary threshold. Additionally, the intersection between VTAs and the deterministic and thresholded probabilistic maps was calculated (VTA_D, VTA_P30, VTA_P40, VTA_P50) and the Dice similarity coefficient (DSC) was defined. To evaluate the clinical significance of each tractography method, a mixed linear model was used with the dependent variable as percent change in CRST scores (% CRST) and the independent variable as volume of overlap. The baseline CRST, age and sex were included as covariates in the model. Our statistical analysis was conducted in SPSS v.29 and a significance level of ≤ 0.05 was used for all tests.

Center of gravity (CG) coordinates for each deterministic and thresholded probabilistic map were calculated using FSL (fslstats). Using the CG coordinates as center point a 4 mm sphere mask was created that corresponded to the CG map for each method. The total Euclidean distance was measured per hemisphere for each patient, and the mean ± standard deviation for each Euclidean distance was calculated. To assess for pairwise differences in the distributions along each axis, a quantitative evaluation of the CG was performed. Specifically, the nonparametric Wilcoxon Signed-Rank test was used since the differences between the pairs of the coordinates between the two methods did not follow a normal distribution. The null hypothesis was that both samples are from the same population. To correct for multiple comparisons, the Bonferroni correction was applied. Finally, to compare the location of the CG and the electrode position, the volume of overlap between the therapeutic VTA and deterministic and probabilistic CG maps was calculated. To evaluate the clinical significance of each tractography method, a Mixed Linear Models was used with %CRST as dependent variable and the volume of overlap as covariate. Also, the age and sex were included in the analysis. To investigate the impact of each tractography method another Mixed Linear Model was run including the interaction effect (volume overlap * tractography method).

As a post-hoc analysis, we used empirical receiver operating characteristic (ROC) curves to examine the sensitivity and specificity of the volumes of overlap that were found significant predictors of improvement in CRST scores. The area under the curve (AUC) and 95 % confidence intervals (CI) were calculated. For classification purposes, the median value of the clinical improvement scores was used as a cut-off value to divide the patients into two groups (superior > median, inferior <= median). Notably, “inferior outcomes” is not meant to imply “non-responders.”

## 3 Results

### 3.1 Processing time

The processing time for probabilistic fiber tracking required approximately 15 h per subject, whereas the total processing time for deterministic fiber tracking required approximately 3 to 5 min per subject.

### 3.2 Comparing deterministic and probabilistic segmentation maps

**Fig. 2:**
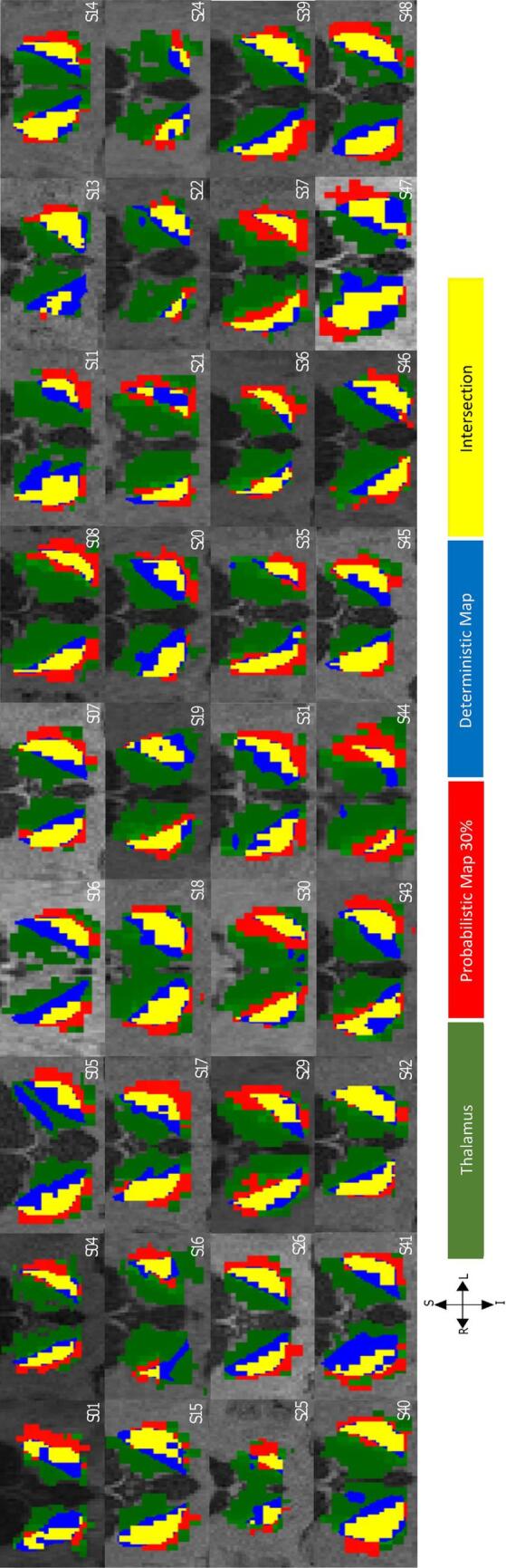
Qualitative comparison between deterministic and probabilistic thalamic segmentation maps. Coronal cross-sections of probabilistic (red) and deterministic (blue) thalamocortical segmentation maps and their intersection (yellow) in T1-weighted space for 36 patients with essential tremor. Both methods delineated the motor region in the posterolateral aspect of the thalamus (green). (For interpretation of the references to color in this figure legend, the reader is referred to the web version of this article.)

Both probabilistic and deterministic methods delineated the region of the thalamus with highest connectivity to the ipsilateral precentral gyrus, which contains the primary motor cortex, for all 36 patients (72 hemispheres). Both tractography methods defined the area of highest connectivity within the thalamus to be within the posterolateral aspect, which is topographically consistent with that previously reported in the literature and with known thalamic anatomy and structural connectivity (Behrens et al., 2003, Traynor et al., 2010). Qualitatively, there was an overlap between deterministic and probabilistic maps (Fig. 2). Quantitatively, the average volume of overlap decreased with greater thresholds of probabilistic maps (30 %: 445 ± 235 mm^3^, 40 %: 365 ± 205 mm^3^, 50 %: 298 ± 165 mm^3^). The DSC results showed that there was a partial overlap between the deterministic and probabilistic maps for all the thresholds (Supplementary Material Fig. 1).

**Fig. 3:**
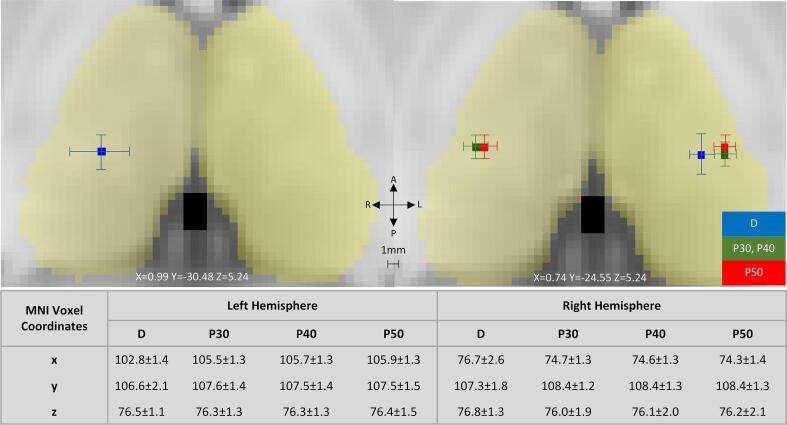
The average center of gravity for deterministic and probabilistic thalamic segmentation maps. A variability between 1 and 3 mm was observed between the deterministic maps (D) and variability of 1 mm was found between the probabilistic maps at 30 % (P30), 40 % (P40) and 50 % (P50) threshold. The average center of gravity of the deterministic maps was found bilaterally more medial posterior compared to the probabilistic maps. (Blue color corresponds to the average center of gravity of D maps. Red color corresponds to average center of gravity of P30 and P40 maps and green to P50 maps). (For interpretation of the references to color in this figure legend, the reader is referred to the web version of this article.)

The mean Euclidean distance between the CG of the two methods increased as the threshold of the probabilistic maps increased (*Left Hemisphere*: DP30 = 3.7 ± 1.3 mm^3^, DP40 = 3.8 ± 1.3 mm^3^ and DP50 = 4.0 ± 1.3 mm^3^, *Right Hemisphere*: DP30 = 3.5 ± 2.2 mm^3^, DP40 = 3.8 ± 2.3 mm^3^ and DP50 = 3.8 ± 2.4 mm^3^). The Wilcoxon Signed-Rank test was used to assess whether the deterministic and probabilistic CG coordinates were significantly different along x, y, and z axes. Along the x axis, the test showed that the difference between the deterministic and the probabilistic maps (for all thresholds) was significant (p < 0.001), with the deterministic CG more medial in both hemispheres. Along the y axis, the distributions on the right hemisphere were significantly different (p < 0.001) between deterministic and probabilistic maps (for all thresholds), while on the left hemisphere the distributions were significantly different (p = 0.002) only for the comparison between deterministic and the 30 % thresholded probabilistic maps. Therefore, bilaterally, the CG of deterministic maps was more posterior compared to probabilistic maps. Finally, along the z axis, the CG was not significantly different between the two methods in both hemispheres. In total, the average CG of deterministic maps was more medial posterior bilaterally when compared to probabilistic maps (Fig. 3**).** The results of this statistical analysis are presented in Table 2.

**Table 2:** Comparison of the medians of the center of gravity between the deterministic and probabilistic maps (P30, P40 and P50) (p < 0.003 after Bonferroni correction).

		**P Wilcoxon Sigh Rank Test**			**Z Wilcoxon Sigh Rank Test**		
		**D vs P30**	**D vs P40**	**D vs P50**	**D vs P30**	**D vs P40**	**D vs P50**
**Left**	x	<0.001	<0.001	<0.001	−5.122	−5.153	−5.169
	y	0.002	0.004	0.008	−3.095	−2.875	−2.655
	z	0.271	0.362	0.561	−1.1	−0.911	−0.581
**Right**	x	<0.001	<0.001	<0.001	−4.587	−4.619	−4.807
	y	<0.001	<0.001	<0.001	−3.582	−3.488	−3.472
	z	0.009	0.018	0.046	−2.608	−2.357	−1.995

**Fig. 4:**
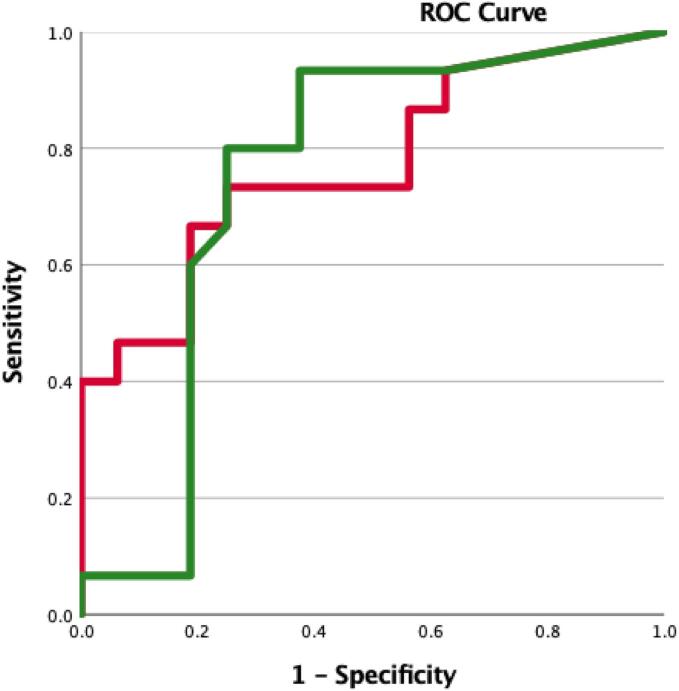
Prediction of clinical improvement. Empirical ROC curves to examine the sensitivity and specificity of the volume of overlaps that were found significant predictors of the improvement in CRST scores. Both methods could reliably predict patients that had superior clinical improvement postoperatively. The AUC was 0.75 (CI:0.56–0.94) for the volume of overlap between the VTA and the deterministically determined CG maps (green) and 0.77 (CI: 0.60–0.94) for the volume overlap between the VTA and the 30% probabilistic CG maps (red). (For interpretation of the references to color in this figure legend, the reader is referred to the web version of this article.)

When the clinical significance of each tractography method was evaluated, the mixed linear model showed that the volume of overlap between the VTA and deterministic (F(1,27) = 5.96, p = 0.02) and probabilistic (F(1,27) = 4.92, p = 0.03) CG maps were a significant predictor of tremor response. When the impact of the tractography method (F(1,57) = 0.58, p = 0.45) was investigated no significant differences was observed. Post-hoc ROC analysis showed that both volumes of overlap between the VTA and deterministic (AUC: 0.75 (CI:0.56–0.94)) and probabilistic (AUC: 0.77 (CI: 0.60–0.94)) CG maps could reliably predict patients that had superior clinical improvement postoperatively (Fig. 4).

## 4 Discussion

### 4.1 Principal findings

Both deterministic and probabilistic tractography methods delineated the region of the thalamus with connectivity to the precentral gyrus to be within the posterolateral aspect of the thalamus. The average CG of deterministic maps was more medial-posterior bilaterally (left hemisphere: 3.7 ± 1.3 mm^3^, right hemisphere: 3.5 ± 2.2 mm^3^) when compared to 30 %-thresholded probabilistic maps. Mixed linear model showed that deterministic and probabilistic (30 %) CG connectivity maps were significant predictors of clinical improvement.

### 4.2 Probabilistic versus deterministic fiber tracking for deep brain stimulation

Several studies have evaluated the use of either the deterministic or probabilistic method for DBS targeting (Riskin-Jones et al., 2021, Gravbrot et al., 2020, Tsolaki et al., 2018, Pouratian et al., 2011, Middlebrooks et al., 2018, Coenen et al., 2014, JM, 2015, Schlaier et al., 2015, Fenoy, 2017, Fenoy and Schiess, 2018, King et al., 2017, Sasada et al., 2017, Chazen et al., 2017, Nowacki et al., 2018, Sudhyadhom et al., 2013, Klein et al., 2012, Akram et al., 2018, Anthofer et al., 2014). However, only a few have directly compared the two methods in this context (Schlaier et al., 2017, Yang et al., 2022, Muller et al., 2019, Petersen et al., 2017). Petersen and colleagues first compared the two tractography methods for the delineation of the hyperdirect pathway between the subthalamic nucleus (STN) and motor cortex, and associated STN target maps in 5 patients undergoing DBS for Parkinson’s disease (Petersen et al., 2017). Whereas the probabilistic method consistently produced a continuous set of connections terminating in the dorsolateral aspect of the STN, the deterministic method reconstructed a comparatively more variable and spurious subset of connections. The target center of gravity between the two methods differed by an average of 1.41 mm. Following up on this study, Muller and colleagues compared the two fiber tracking methods for the delineation of sensorimotor regions in the STN and globus pallidus internus (GPi) in 7 patients with refractory PD selected for DBS (Muller et al., 2019). Although both tracking methods defined the sensorimotor region within the posterosuperolateral aspect of the ipsilateral STN and within the posterolateral aspect of the GPi, the deterministic method again reconstructed smaller and more variable streamlines. Overall, the center of gravity between the two methods differed by an average of 2.67 mm. Most recently, Yang and Parker compared deterministic and probabilistic fiber tracking of the dentato-rubro-thalamic tract (DRTT) in 19 patients with ET who underwent DBS (Yang et al., 2022). The authors found that the probabilistic-derived DRTT (3.32 ± 1.70 mm) was closer to the clinically optimal active contacts than the DRTT derived by deterministic methods (5.01 ± 2.12 mm). However, this study did not directly assess whether the relationship between the DBS electrode and fiber tracts was associated with degree of tremor reduction. In our study, we found that both deterministic and probabilistic tractography methods were able to consistently identify robust connections between the ipsilateral motor cortex and thalamus. The average center of gravity of deterministic target maps was more medial posterior bilaterally when compared to probabilistic target maps. Whereas volume of overlap between VTAs and target maps were not predictive of tremor response, overlap between VTAs and the center of gravity of deterministic and probabilistic (30 % threshold) targets maps were reliably predictive of improvement.

Disparities in results between the aforementioned studies may potentially be explained by the difference in anatomical size of the ROIs used in the analyses. Whereas the thalamus is approximately 20 mm in width (Lierse, 1993), the STN and GPi measure approximately 4–5 mm (Schaltenbrand and Wahren, 1998), likely making it more difficult to capture the tracts arising from these grey matter structures. Moreover, evidence suggests that the complexities of the tracts themselves may affect the ability to reconstruct pathways using the deterministic method. Consistent with our findings, Behrens and colleagues found that connectivity-based segmentation of thalamus was largely unchanged between single and multi-fiber approaches, suggesting that the same dominant pathway is found by both approaches (Behrens et al., 2007). Schlaier and colleagues compared the two tractography approaches for identifying cerebellar-thalamic fiber bundles in six patients (12 hemispheres) with movement disorders (five with PD and one with ET) (Schlaier et al., 2017). They concluded that probabilistic tractography is more sensitive and robust than the deterministic method for detecting the DRTT and adjacent relevant fiber tracts such as the ansa lenticularis. Nevertheless, they showed that 12-direction deterministic fiber tracking (FT) was still able to produce DRTT fiber tracks in 9/12 hemispheres; 8 with complete overlap and 1 with partial overlap in comparison to DRTT fiber tracks produced by 64-direction probabilistic tractography. Additionally, when attempting to delineate cerebellothalamocortical (CTC) tracts, the authors found that deterministic FT was significantly more often successful in detecting the CTC than in detecting the DRTT. The authors suggested that this advantage in sensitivity was likely due to the fact the CTC and its ROIs are constrained to the ipsilateral hemisphere and that this approach avoids decussating fibers at the level of the diencephalon. Yang and Parker found that in a total of 32 implanted hemispheres, probabilistic tractography successfully reconstructed the decussating DRTT and the non-decussating DRTT (Meola et al., 2016) in all hemispheres. By contrast, deterministic tractography reconstructed the decussating DRTT in only 11 of the 32 hemispheres (31 %), whereas it was able to reconstruct the non-decussating DRTT in all hemispheres (Yang et al., 2022). By means of a deterministic algorithm, Sammartino and colleagues utilized ipsilateral ROIs (i.e. the cerebral peduncle, primary motor cortex, dorsal column, and primary sensory cortex) to define the lateral and posterior borders of the Vim by tracking the pyramidal tract and medial lemniscus, which are typically considered major tracts that are easily trackable (Sammartino et al., 2016). Krishna and colleagues then prospectively utilized this deterministic-based method for focused ultrasound thalamotomy in 10 ET patients (Krishna et al., 2019). The authors concluded that their tractography-based targeting method was both safe and clinically effective in the short-term. Additionally, a post hoc thalamic connectivity analysis demonstrated that the location of the thalamotomy lesion significantly overlapped with the Vim defined by a probabilistic tractography-based thalamic segmentation. In total, the evidence suggests that the deterministic method can perform comparably to the probabilistic method when larger ROIs and less complex pathways are of interest.

### 4.3 Clinical implications

In the past decade, there has been an abundance of evidence demonstrating utilization of DT as a direct targeting approach for DBS (Calabrese, 2016, Middlebrooks et al., 2020, Gravbrot et al., 2020), with multiple studies suggesting that probabilistic connectivity-based segmentation of the thalamus is predictive of improvement in ET, particularly when connected to nodes in the motor network (Tsolaki et al., 2018, Pouratian et al., 2011, Middlebrooks et al., 2018, Behrens et al., 2003, Riskin-Jones et al., 2021). Personalized DBS targeting with DT may offer potential benefits including improved target accuracy with fewer electrode passes and consequently, better outcomes and less adverse events. Additionally, it may allow expansion of DBS therapy to additional indications where target accuracy and lack of a network-based targeting approach have historically been obstacles to achieving success (Kashanian et al., 2021). However, one of the major barriers towards implementation of the probabilistic approach to the clinical setting is the fact that this fiber tracking algorithm is both computationally and time intensive, leading many to suggest the need for more practical yet effective alternative methods. Whereas the probabilistic method has demonstrated greater sensitivity for identifying complex fiber pathways, the results of our study further demonstrate that the deterministic method may be suitable for tracking less complex, dominant fiber pathways (Behrens et al., 2007). Although deterministic target maps did not exactly replicate probabilistic maps, these differences are likely not clinically significant given DBS electrode geometry and size of typical stimulation field models.

Consistent with previous studies (Schlaier et al., 2017, Muller et al., 2019, Petersen et al., 2017), the deterministic method required substantially less time for analysis, requiring an average of 3–5 min on a standard computer compared to the 15 h needed on a parallel processing unit for the probabilistic method. Moreover, the deterministic fiber tracking pipeline was completely automated and fully integrated into a neuronavigation software suite that many neurosurgeons are already accustomed to. Using this tractography method will encourage utilization of connectivity-based thalamic targeting in the clinical setting and may further aid in our understanding of DBS mechanisms.

### 4.4 Limitations and future directions

Our study provides the first comparison between deterministic and probabilistic fiber tracking for deriving connectivity-based motor-thalamic target regions in the context of DBS for patients with ET. Our study evaluated the two tracking algorithms in a total of 36 patients (72 hemispheres); the most patients evaluated on this topic. Moreover, our study is a follow-up to published studies which have already demonstrated that probabilistic connectivity-based segmentation of the thalamus correlates with clinical improvement in ET, especially when connected to cortical motor areas (Pouratian et al., 2011, Middlebrooks et al., 2018). Since we had already validated the probabilistic tractography method, our goal was to evaluate how closely the deterministic method approaches the probabilistic method.

Although both tractography methods delineated the connectivity-based target within the posterolateral region of the thalamus, we observed some variability in percent volume of overlap and center of gravity coordinates between and within subjects. Additionally, when comparing the tractography methods, we consistently observed slightly greater percent volume of overlap and less average distance between centers of gravity in the right hemisphere. Middlebrooks and colleagues reported a high level of intra- and inter-subject variability in probabilistic tractography-based thalamic segmentations in 32 subjects with ET (Middlebrooks et al., 2018). When comparing indirect target maps to their probabilistic-based segmentations, they found that the indirect target generally encompassed more of the ventralis oralis nucleus on the left hemisphere and more of the Vim on the right. Interestingly, a large study of left versus right hemisphere differences in brain connectivity in 569 twins identified significant differences in the proportions of fibers intersecting left and right hemisphere cortical regions, and an increase in the relative fiber density favoring the right hemisphere with age (Daianu et al., 2012). Whether the observed asymmetry in our study was due to real anatomical differences in structural connectivity or technical limitations will require further investigation.

In the current study, deterministic-based thalamic segmentation maps were created using a fully automated analysis pipeline and default fiber tracking parameters. Greater levels of anatomical accuracy may be achieved by refining tractography parameters, such as the angular threshold, but that was beyond the scope of this primary analysis (Thomas et al., 2014). Furthermore, electric field modeling based on stimulation parameters may also help improve accuracy by providing an estimation of the area being modulated. Evidence suggests that the deterministic method can potentially be used to delineate major fibers of the DRTT for DBS targeting if ROIs are selected appropriately (Schlaier et al., 2017, Yang et al., 2022, Sammartino et al., 2016). Future prospective studies will be important to determine whether tract or nuclear stimulation yield the best clinical outcome. Using more advanced deterministic tractography algorithms which can resolve crossing fibers may also help further narrow any incongruence between the methods and increase the accuracy of defining our target region. Ultimately, we believe the results of our study show promise of a practical and reliable method for clinical implementation of connectivity-based DBS targeting, thereby setting the stage for evaluation in future prospective clinical trials.

## 5 Conclusions

This is the first comparison study between deterministic and probabilistic fiber tracking for deriving connectivity-based thalamic motor regions for DBS in patients with ET. The results suggest that when used appropriately, deterministic fiber tracking can reconstruct thalamic target maps for DBS that are comparable to those produced by probabilistic methods. Comparability of deterministic and probabilistic methods however should not be generalized across targets. Our study represents another step further in the direction of implementing tractography-based targeting into the clinical setting.

## Funding

This research did not receive any specific grant from funding agencies in the public, commercial, or not-for-profit sectors.

## CRediT authorship contribution statement

**Evangelia Tsolaki:** Writing – review & editing, Writing – original draft, Software, Methodology, Formal analysis, Data curation, Conceptualization. **Alon Kashanian:** Writing – review & editing, Writing – original draft, Software, Investigation, Formal analysis, Data curation, Conceptualization. **Kevin Chiu:** Writing – original draft, Methodology. **Ausaf Bari:** Writing – review & editing, Supervision. **Nader Pouratian:** Writing – review & editing, Supervision, Conceptualization.

## Declaration of competing interest

The authors declare the following financial interests/personal relationships which may be considered as potential competing interests: Dr. Pouratian and Dr. Bari report grants and personal fees from Brainlab.

## Data Availability

Data will be made available on request.
