# Supplementary material for: Connectivity-based segmentation of the thalamic motor region for deep brain stimulation in essential tremor: A comparison of deterministic and probabilistic tractography
Source: Neuroimage Clin. 2024 Feb 27;41:103587. doi: 10.1016/j.nicl.2024.103587 (PMC10944185; doi:10.1016/j.nicl.2024.103587)
Supplement: Supplementary Data 1 [file mmc1.docx]

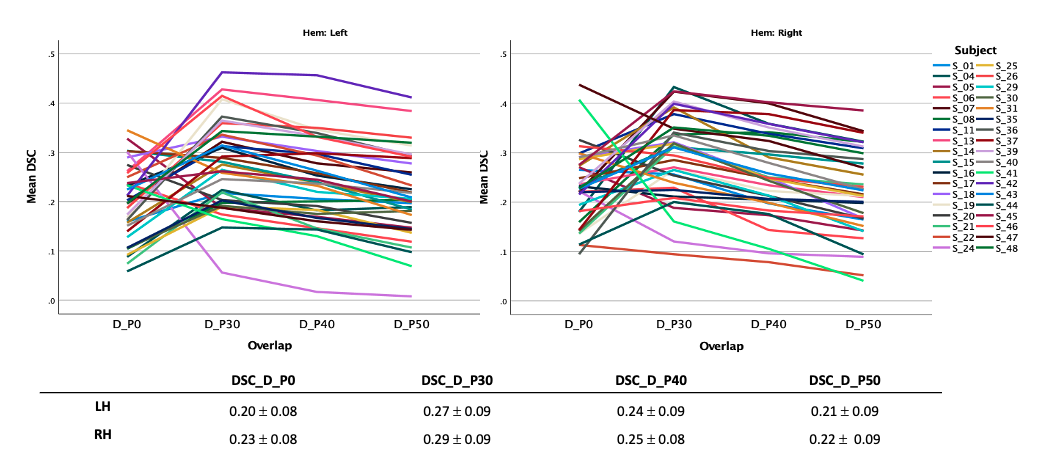


***Supplementary Material Figure*** *1. The Dice similarity coefficient (DSC) was calculated to evaluate the overlap between the deterministic and probabilistic maps at different thresholds. Considering that DSC ranges from 0–1, with a score of 0 indicating no overlap and a score of 1 indication perfect overlap, the results showed that there was a partial overlap between the deterministic and probabilistic maps for all the thresholds.*
